# Vitamin D Deficiency Is Associated with Impaired Sensitivity to Thyroid Hormones in Euthyroid Adults

**DOI:** 10.3390/nu15173697

**Published:** 2023-08-24

**Authors:** Liyuan Zhou, Ying Wang, Jingru Su, Yu An, Jia Liu, Guang Wang

**Affiliations:** 1 Department of Endocrinology, Beijing Chao-Yang Hospital, Capital Medical University, Beijing 100020, China; zhouliyuan_mail@163.com (L.Z.); sujingru97@163.com (J.S.); anyu900222@126.com (Y.A.); liujiacy@ccmu.edu.cn (J.L.); 2 Medical Examination Center, Beijing Chao-Yang Hospital, Capital Medical University, Beijing 100020, China; wangying1@bjcyh.com

**Keywords:** vitamin D, thyroid hormone sensitivity, thyroid function, euthyroid adults

## Abstract

The relationship between vitamin D deficiency and sensitivity to thyroid hormones was unclear. We aimed to explore the association of 25-hydroxyvitamin D (25(OH)D) levels with thyroid hormone sensitivity in euthyroid adults. A total of 3143 subjects were included. The serum 25(OH)D, free thyroxine (FT3), free thyrotropin (FT4), thyroid-stimulating hormone (TSH), and other clinical variables were measured. Vitamin D deficiency was defined as 25(OH)D < 20 ng/mL. Thyroid feedback quantile-based index (TFQI), parametric thyroid feedback quantile-based index (PTFQI), thyroid-stimulating hormone index (TSHI), thyrotrophic thyroxine resistance index (TT4RI), and FT3/FT4 were calculated to assess thyroid hormone sensitivity. Results showed that 58.8% of the participants had vitamin D deficiency. They had significantly higher levels of triglyceride, insulin, FT3, FT4, TSH, TFQI, PTFQI, TSHI, and TT4RI and lower levels of high-density lipoprotein cholesterol than those with sufficient vitamin D (all *p* < 0.05). Logistic regression analysis showed that the risk of impaired sensitivity to thyroid hormones evaluated by TFIQ, PTFQI, TSHI, and TT4RI increased by 68% (OR: 1.68; 95%CI: 1.45–1.95; and *p* < 0.001), 70% (OR: 1.70; 95%CI: 1.46–1.97; and *p* < 0.001), 66% (OR: 1.66; 95%CI: 1.43–1.92; and *p* < 0.001), and 50% (OR: 1.50; 95%CI: 1.30–1.74; and *p* < 0.001), respectively, in participants with vitamin D deficiency compared with those with sufficient vitamin D after adjusting for multiple confounders. In conclusion, in euthyroid populations, vitamin D deficiency was associated with impaired sensitivity to thyroid hormones.

## 1. Introduction

Vitamin D deficiency has become a public health problem, and it is especially highly prevalent in the Middle East and Asia [1]. Vitamin D is a type of fat-soluble hormone and is influenced by age, race, sun exposure, food intake, and adiposity [2]. Serum 25-hydroxyvitamin D (25(OH)D) is the best indicator of vitamin D status. The well-known function of vitamin D is associated with the regulation of calcium (Ca) and phosphorus (P) metabolism and bone growth [2]. Additionally, studies uncovered the widespread distribution and expression of the vitamin D receptor and its related metabolic enzymes in fat, pancreas, pituitary, and thyroid cells, indicating vitamin D’s pleiotropic functions in health and diseases [3]. Previous studies have demonstrated that low 25(OH)D levels are related to infection [4,5], autoimmune diseases [6,7], and endocrine metabolic disorders such as diabetes [8], adrenal diseases [9], and polycystic ovary syndrome [10]. Recent evidence indicated a relationship between vitamin D concentrations and thyroid diseases, including autoimmune thyroid disease and thyroid cancer [11]. However, the relationship between vitamin D and thyroid hormones is largely unclear and controversial. Observational studies showed either positive [12], negative [13], or no associations [14] of 25(OH)D with thyroid hormones in healthy populations. Even in patients with autoimmune thyroid diseases (ALTD), some studies found a negative relationship between thyroid-stimulating hormone (TSH) and 25(OH)D, and others did not observe associations [15]. Variations in thyroid hormone sensitivity might partially explain these inconsistencies.

During the past few years, the association of sensitivity to thyroid hormones with metabolic health received increasing attention. In 2019, Laclaustra et al. firstly proposed the thyroid feedback quantile-based index (TFQI) to evaluate the central sensitivity to thyroid hormones and showed that central resistance to thyroid hormones was associated with increased risks of obesity, diabetes, and metabolic syndrome [16]. Moreover, researchers proposed other indices assessing central sensitivity to thyroid hormones, including the parametric thyroid feedback quantile-based index (PTFQI) [16], thyrotrophic thyroxine resistance index (TT4RI), and thyroid-stimulating hormone index (TSHI) [17]. Subsequently, Teng et al. demonstrated that impaired sensitivity to thyroid hormones was related to higher risks of cardiovascular disease and hyperuricemia and a lower risk of obesity in patients with subclinical hypothyroidism [18]. Recently, the association of impaired sensitivity to thyroid hormones with high levels of homocysteine [17], components of metabolic syndrome [19], and non-alcoholic fatty liver disease [20] were also revealed. However, the influencing factors of sensitivity to thyroid hormones and whether vitamin D deficiency is correlated with reduced sensitivity to thyroid hormones are unknown. In this study, we aimed to investigate the association of vitamin D deficiency with thyroid hormone sensitivity in an euthyroid adult population in Beijing, China.

## 2. Materials and Methods

### 2.1. Study Design and Populations

This was a single-center population-based cross-sectional study. Participants aged over 18 who performed routine health check-ups in Beijing Chao-yang Hospital from April 2016 to August 2021 were recruited. All participants lived in Beijing and received tests of metabolic biomarkers and thyroid-associated variables. Subjects who met the following criteria were excluded: abnormal thyroid function or with the history of thyroid diseases, antithyroid therapy, and hormone replacement treatment; severe hepatic dysfunction or renal dysfunction; or using vitamin D supplements (Figure S1). Finally, 3143 individuals were included in the data analyses. All subjects gave their informed consent for inclusion before they participated in the study. The study was conducted in accordance with the Declaration of Helsinki, and the protocol was approved by the Ethics Committee of the Beijing Chao-yang Hospital affiliated with Capital Medical University (2022-KE-517).

### 2.2. Clinical Variable Measurements

All subjects underwent anthropometric measurements, including age, sex, height, body weight, and blood pressure by certified clinical doctors. Systolic blood pressure (SBP) and diastolic blood pressure (DBP) were measured three times consecutively using a standard sphygmomanometer. The mean values of the three recorded blood pressure were used for data analyses. 

After 10 h of overnight fasting, peripheral blood samples were collected for laboratory tests. Fasting blood glucose, total cholesterol (TC), triglyceride (TG), high-density lipoprotein cholesterol (HDL-C), low-density lipoprotein cholesterol (LDL-C), lipoprotein (a) (Lp(a)), alanine transaminase (ALT), aspartate transaminase (AST), Ca, P, urea, creatinine (Cr), and uric acid (UA) were measured by routine automated laboratory methods ((Hitachi 7060C automatic biochemistry analysis system) in Beijing Chao-yang Hospital. Hemoglobin A1c (HbA1c) was assessed by high-performance liquid chromatography (Huizhong MQ-6000, Shanghai, China). Fasting insulin levels were measured by radioimmunoassay. Serum 25(OH)D, TSH, free thyroxine (FT3), free thyrotropin (FT4), antithyroglobulin (Anti-TG), and antithyroidperoxidase (Anti-TPO) were measured using an electrochemiluminescence immunoassay in Beijing Chao-yang Hospital. 

### 2.3. Calculations and Definitions

Body mass index (BMI) was calculated as body weight in kilograms divided by height in meters squared (kg/m^2^). Insulin resistance was assessed by the homeostasis model for insulin resistance (HOMA-IR), calculated as fasting insulin (mIU/L) × fasting blood glucose (mmol/L)/22.5. Homeostasis model β (HOMA-β) was used to assess insulin secretion and was calculated as 20 × fasting insulin (mIU/L)/(fasting blood glucose [mmol/L] − 3.5). Homeostatic model for insulin sensitivity index (HOMA-ISI) was calculated as 1/(fasting blood glucose [mmol/L] × fasting insulin [mIU/L]). TyG index, AIP, non-HDL-C, and RC were calculated according to following equations: TyG (mg/mL)^2^ = ln [fasting plasma glucose (mg/dL) × TG (mg/mL)/2]; AIP = log (TG/HDL-C); non-HDL-C = TC − HDL-C; and RC = non-HDL-C − LDL-C.

Several different indices were calculated to evaluate sensitivity to thyroid hormones. TFQI was calculated as cdf FT4 − (1 − cdf TSH) [16]. TSHI was calculated as ln TSH (mIU/L) + 0.1345 × FT4 (pmol/L). TT4RI was defined as FT4 (pmol/L) × TSH (mU/L) [18]. We also calculated PTFQI by the standard normal cumulative distribution function: Φ ((FT4-μFT4)/σFT4) − (1 − Φ ((Ln TSH − μLn TSH)/σLn TSH)). In this population, μFT4 was 16.5545, σFT4 was 1.9925, μLn TSH was 0.6347, and σLn TSH was 0.4513. The value of TFQI and PTFQI ranged from −1 to 1. Positive values indicated poor central sensitivity to thyroid hormones, whereas negative values suggested good thyroid hormone sensitivity. As for TSHI and TT4RI, the higher the values the lower the central thyroid hormone sensitivity. Peripheral sensitivity to thyroid hormone was assessed by FT3/FT4 ratio. Lower FT3/FT4 indicated reduced peripheral sensitivity to thyroid hormones. The normal range of thyroid function in this study was TSH (0.55–4.78 μIU/mL), FT3 (3.54–6.47 pmol/L), and FT4 (11.45–22.65 pmol/L). Vitamin D deficiency was defined as 25(OH)D level < 20 ng/mL [21]. Impaired sensitivity to thyroid hormones was defined as TFQI, PTFQI, TSHI, and TT4RI larger or FT3/FT4 smaller than their respective mean values in this population.

### 2.4. Statistical Analysis

In this study, the subjects were categorized based on serum 25(OH)D levels. The normality of the data was assessed using normal P-P plots. Normally distributed continuous variables or one-way ANOVA with Tukey’s post hoc test were expressed as mean ± standard deviation and were compared using Student’s *t*-tests. Non-normally distributed continuous variables were expressed as median and interquartile range (median (IQR25-IQR75)) and were compared using Student’s *t*-tests or one-way ANOVA with Tukey’s post hoc test after natural log-transformed. Categorical variables were compared using Fischer’s exact tests. When performing correlation, non-normally distributed continuous variables were natural log transformed. Pearson’s and partial correlation coefficients were used to explore the association of 25(OH)D with thyroid-associated variables. To further evaluate the potential associations of vitamin D deficiency with impaired sensitivity to thyroid hormones, a logistic regression model was performed. A two-tailed *p* < 0.05 was considered statistically significant. SPSS 22.0 was used for statistical analysis.

## 3. Results

### 3.1. Clinical Characteristics of the Population

**Figure 1:**
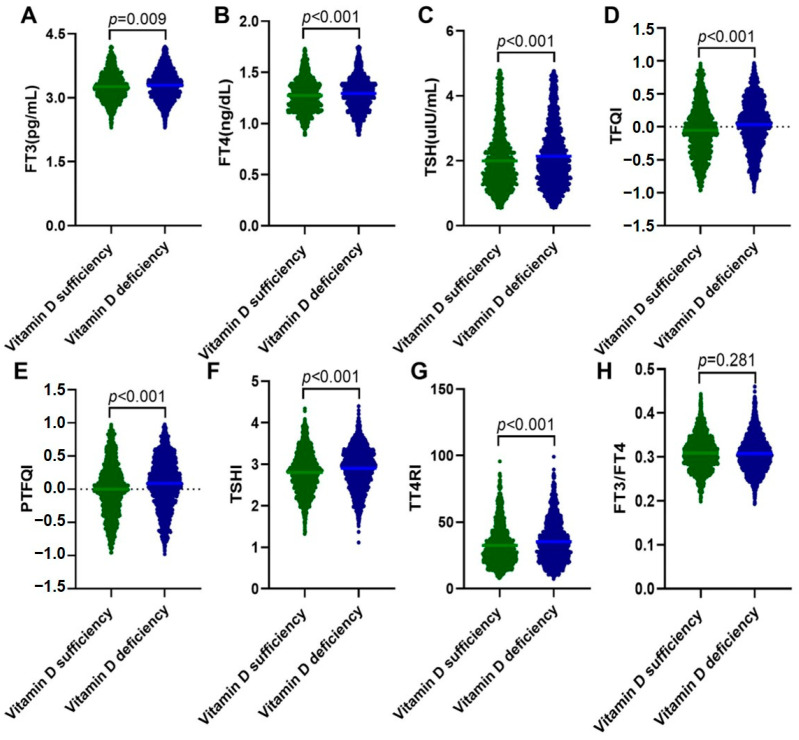
Levels of thyroid-associated variables in the vitamin D sufficiency group and the vitamin D deficiency group. Data were analyzed by Student’s *t*-tests. (**A**) FT3; (**B**) FT4; (**C**) TSH; (**D**) TFQI; (**E**) PTFQI; (**F**) TSHI; (**G**) TT4RI; and (**H**) FT3/FT4. FT3, free triiodothyronine; FT4, free thyrotropin; TSH, thyroid-stimulating hormone; TFQI, thyroid feedback quartile-based index; PTFQI, parametric thyroid feedback quantile-based index; TSHI, TSH index; and TT4RI, thyrotropin thyroxine resistance index.

Among 3143 euthyroid adults finally included in this study, 1849 (58.8%) had vitamin D deficiency (Table 1). They presented significantly higher levels of TG (*p* < 0.001), non-HDL-C (*p* = 0.012), AIP (*p* < 0.001), insulin (*p* = 0.007), HOMA-IR (*p* = 0.018), HOMA-β (*p* = 0.028), and TyG index (*p* < 0.001) and lower levels of HDL-C (*p* = 0.014), Ca (*p* = 0.011), and HOMA-ISI (*p* = 0.018) than those with sufficient vitamin D. For thyroid-associated variables, although within the normal ranges, FT3, FT4, and TSH levels were significantly higher in the vitamin D deficiency group than the vitamin D sufficiency group (all *p* < 0.001). The percentage of Anti-TG and Anti-TPO positivity was matched between the two groups. However, central thyroid hormone sensitivity indices, including TFQI, PTFQI, TSHI, and TT4RI, were all significantly elevated in participants with vitamin D deficiency (all *p* < 0.001), whereas the peripheral thyroid hormone sensitivity index FT3/FT4 showed no differences between the two groups (Figure 1). As vitamin D deficiency and thyroid dysfunction are more common in women, we further compared the clinical characteristics between men and women (Table S1). The results showed that 673 (59.5%) women and 1176 (58.4%) men had vitamin D deficiency. Interestingly, women with vitamin D deficiency presented lower FT3 and FT4 and higher TSH and anti-TG and anti-TPO positivity than men (all *p* < 0.001). They also had significantly elevated levels of TFQI, PTFQI, TSHI, and TT4RI and reduced FT3/FT4 levels.

### 3.2. 25(OH)D Levels Were Significantly Correlated with Thyroid Hormone Sensitivity Indices

Then, we analyzed the correlations between 25(OH)D levels and thyroid-associated variables (Table 2). 25(OH)D was significantly negatively correlated with FT3 (*p* = 0.022), FT4 (*p* = 0.006), TSH (*p* < 0.001), and thyroid hormone sensitivity indices such as TFQI, PTFQI, TSHI, and TT4RI (all *p* < 0.001) both before and after adjusting for age, sex, and BMI. Even after further adjusting for other potential confounders, including DBP, TG, glucose, ALT, Cr, and UA, the relationship between 25(OH)D and thyroid hormone sensitivity indices were still statistically significant in the whole population and in women (Table S2).

### 3.3. Vitamin D Deficiency Was Associated with Markedly Increased Risks of Impaired Sensitivity to Thyroid Hormones

**Figure 2:**
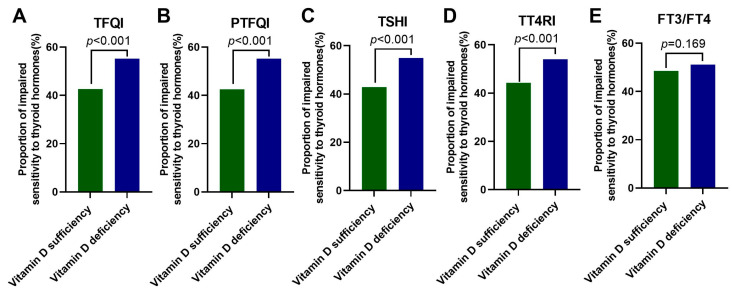
The proportions of participants with impaired sensitivity to thyroid hormones in the vitamin D sufficiency group and the vitamin D deficiency group. Data were analyzed by Fisher’s exact test. (**A**) TFQI; (**B**) PTFQI; (**C**) TSHI; (**D**) TT4RI; and (**E**) FT3/FT4. TFQI, thyroid feedback quartile-based index; PTFQI, parametric thyroid feedback quantile-based index; TSH, thyroid-stimulating hormone; TSHI, TSH index; TT4RI, thyrotropin thyroxine resistance index; FT3, free triiodothyronine; and FT4, free thyrotropin.

Next, we compared the proportions of participants with impaired sensitivity to thyroid hormones between the vitamin D sufficiency group and the vitamin D deficiency group for each sensitivity index (Figure 2A–E). The results showed that higher percentages of subjects in the vitamin D deficiency group developed impaired central sensitivity to thyroid hormones evaluated by TFQI, PTFQI, TSHI, and TT4RI compared with those in the vitamin D sufficiency group (all *p* < 0.001), which was more evident in women (Figure S2).

**Figure 3:**
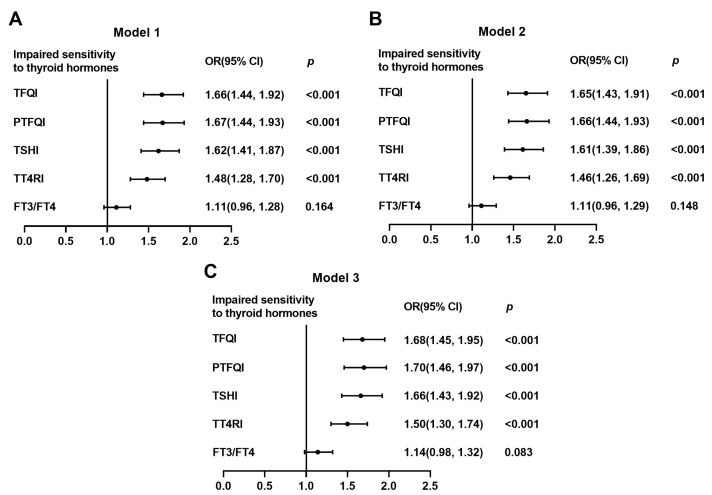
Logistic analysis of the association between vitamin D deficiency and impaired sensitivity to thyroid hormones. (**A**) Model 1, unadjusted; (**B**) Model 2, adjusted for age, sex, and BMI; and (**C**) Model 3, adjusted for age, sex, BMI, DBP, TG, glucose, ALT, Cr, and UA. Data were analyzed by logistic regression analysis. OR, odds ratio; CI, confidence interval; TFQI, thyroid feedback quartile-based index; PTFQI, parametric thyroid feedback quantile-based index; TSH, thyroid-stimulating hormone; TSHI, TSH index; TT4RI, thyrotropin thyroxine resistance index; FT3, free triiodothyronine; FT4, free thyrotropin; BMI, body mass index; DBP, diastolic blood pressure; TG, triglyceride; ALT, alanine aminotransferase; Cr, creatinine; and UA, uric acid.

Moreover, to investigate the relationship between vitamin D deficiency and impaired sensitivity to thyroid hormones, we performed logistic regression analyses (Table 3). The risk of impaired sensitivity to thyroid hormones, evaluated by TFIQ, PTFQI, TSHI, and TT4RI, increased by 66% (OR: 1.66; 95%CI: 1.44–1.92; and *p* < 0.001), 67% (OR: 1.67; 95%CI: 1.44–1.93; and *p* < 0.001), 62% (OR: 1.62; 95%CI: 1.41–1.87; and *p* < 0.001), and 48% (OR: 1.48; 95%CI: 1.28–1.70; and *p* < 0.001), respectively, in participants with vitamin D deficiency compared with those with sufficient vitamin D. Even after adjusting for age, sex, BMI, DBP, TG, glucose, ALT, Cr, and UA, the associations between vitamin D deficiency and impaired sensitivity to thyroid hormones were not changed (Figure 3). Although similar patterns were identified in men and women when we did multivariate logistic regression analysis using the same adjusting for confounder except for sex, the ORs of impaired sensitivity to thyroid hormones were significantly higher in women than men in individuals with vitamin D deficiency ([TFQI: 2.84 vs. 1.25; PTFQI: 2.92 vs. 1.25; TSHI: 2.86 vs. 1.24; TT4RI: 2.40 vs. 1.18; and all *p* for interaction < 0.001) (Table 4).

## 4. Discussion

In this euthyroid adult population, we demonstrated that participants with vitamin D deficiency were more susceptible to metabolic disorders and impaired sensitivity to thyroid hormones. Among them, women had higher positive rates of thyroid autoantibodies and poor thyroid hormone sensitivity than men. Vitamin D deficiency was significantly associated with impaired sensitivity to thyroid hormones (increased TFQI, PTFQI, TSHI, and TT4RI) even after adjusting for multiple confounding factors, especially in women.

Vitamin D deficiency has been a public health problem. In the Endocrine Society’s Practice Guidelines, 25(OH)D < 20 ng/mL was defined as vitamin D deficiency [21]. Based on this cutoff, it has been reported that approximately 30% of children and adults worldwide have vitamin D deficiency. Consistently, Robin et al. showed that 31% of Australian adults aged 25 years and older were vitamin D deficient [22]. The prevalence of low levels of vitamin D was highest in Asia, the Middle East, and Africa [23]. An epidemiological study of 8976 participants aged over 59 years in Korea reported that 59.7% of men and 86.5% of women had a serum 25(OH)D level < 20 ng/mL [24]. In China, low vitamin D status was also common. Liu et al. measured the serum 25(OH)D level in 2493 elderly individuals aged 65–112 years from eight centers of China and demonstrated that 41.3% of them were vitamin D deficient [25]. A cross-sectional study among 14,302 subjects aged 18–65 years from six cities in China showed the rate of vitamin D deficiency was 50.3% [26], whereas another cross-sectional study performed in eight cities of China reported as high as 80.6% of the adult participants had a serum 25(OH)D level < 20 ng/mL [27]. Zhen et al. showed a vitamin D deficiency rate of 75.2% in middle-aged and elderly participants in northwestern China [28]. In the present study, the average value of 25(OH)D was 19.30 ng/mL. In total, 58.8% of the adult population from Beijing, one of the northernmost cities of China, had vitamin D deficiency. The high heterogeneity regarding the prevalence of vitamin D deficiency might be attributed to differences in age, sex, race, geographic area, sun exposure, and outdoor physical activity among the published studies. Nevertheless, all these studies indicate that vitamin D deficiency has become a global pandemic and needs more evaluation, prevention, and treatment. 

It has been well-acknowledged that vitamin D plays essential roles in calcium and phosphorus metabolism and bone mineral balance. Nevertheless, with the disclosure of vitamin D functions, despite limited and inconsistent, emerging studies uncovered the role of vitamin D deficiency in metabolic health. A study designed to investigate the association of vitamin D status with cardiovascular metabolic risk factors in healthy individuals suggested that serum 25(OH)D concentrations were negatively related to multiple metabolic risk factors, including SBP and serum LDL-C levels, regardless of BMI in male subjects [29]. Reduced insulin sensitivity and β cell function and a higher LDL-C to HDL-C ratio were observed in subjects with vitamin D deficiency in studies performed by Chiu et al. [30] and by Carbone et al. [31]. Lin et al. firstly analyzed the relationship between plasma 25(OH)D levels and metabolic disorders in Chinese individuals aged 50–70 years and showed that the 25(OH)D level was significantly inversely associated with metabolic syndrome components and HbA1c after adjusting for multiple factors, whereas the association of plasma 25(OH)D with triglycerides and HDL-C was only observed in men but not in women [32]. In this study, we found that participants with vitamin D deficiency had higher levels of TG, AIP, TyG, insulin, and insulin resistance index and lower HDL-C. 

In addition to bone metabolism and metabolic health, emerging evidence showed that vitamin D deficiency also correlated with thyroid diseases, especially AITD and thyroid carcinoma [15]. Nevertheless, clinical evidence linking 25(OH)D level to thyroid function is limited and conflicting. A study performed among euthyroid adults showed a strong positive association of vitamin D deficiency with TSH levels after adjusting for age, gender, and season [33]. Another study also demonstrated a negative relationship between 25(OH)D levels and TSH in patients with hypothyroidism [34]. But, no relations were observed in a U.S. population [35] and in healthy children aged 6 to 24 months [36] between 25(OH)D and thyroid hormones. Even results of studies investigating the effects of vitamin D supplementation on TSH and thyroid hormone were also inconsistent. Some study noted a decrease in TSH and thyroid hormone but others found no changes [15]. The largest-scale study with 11,017 participants showed a significant reduction in TSH and thyroid hormone after 12 months vitamin D supplementation [37]. Our study showed that serum FT4, FT4, and TSH were higher in subjects with vitamin D deficiency than those with sufficient vitamin D, and serum 25(OH)D levels were significantly negatively correlated with FT3, FT4, and TSH levels in euthyroid adults even after adjusting for all potential confounders. Although differences in race, sample size, healthy status, sun exposure, and some other confounders may partially explain the inconsistencies, we identified that serum 25(OH)D levels were inversely correlated with TFQI, PTFQI, TSHI, and TT4RI, indicating that vitamin D deficiency was associated with impaired sensitivity to thyroid hormones. 

There is a negative feedback loop between thyroid hormones and TSH physiologically. However, high levels of serum thyroid hormones and TSH levels coexisted in patients with resistance to thyroid hormone syndrome caused by thyroid hormone receptor mutations [38]. Thyroid hormone resistance to compensate for homeostasis also exists [39]. Laclaustra et al. firstly proposed the TFQI by applying population empirical cumulative distribution function (cdf) to hormone concentrations to evaluate the sensitivity to thyroid hormones in 2019. The lower the values, the higher the sensitivity. They showed that higher levels of TFQI were associated with increased risks of obesity, diabetes, and metabolic syndrome in a U.S. population [16]. Subsequently, Nie et al. found that higher serum adipocyte fatty acid-binding protein levels were associated with reduced sensitivity to thyroid hormones in a euthyroid population [40]. Another study confirmed that TFQI was the indicator of resistance to thyroid hormones and was associated with diabetes and hypertension in euthyroid Tehranian populations [41]. Recently, emerging studies investigated the relationship between sensitivity to thyroid hormones, metabolic disorders, and non-alcoholic fatty liver disease in Chinese subjects [18,19,20,42]. However, the influencing factors of thyroid hormone sensitivity is unclear. Our study showed that vitamin D deficiency was significantly associated with reduced sensitivity to thyroid hormones (increased TFQI, PTFQI, TSHI, and TT4RI) for the first time. Even after adjusting for confounders, including age, sex, BMI, and other metabolic parameters, the risks of reduced sensitivity to thyroid hormones still increased by more than 50% in participants with vitamin D deficiency. Furthermore, given the phenomenon that thyroid dysfunction is more common in women than men [43], and vitamin D deficiency is more common in women with thyroid diseases [44], we further investigated this relationship in women and men, respectively. Consistently, our results showed that the relationship between vitamin D deficiency and impaired sensitivity to thyroid hormones was significantly closer and stronger in women than men and implicated that women were more susceptible to impaired sensitivity to thyroid hormones in patients with vitamin D deficiency.

The specific mechanisms underlying the association of 25(OH)D levels with thyroid function and thyroid hormone sensitivity are mysterious. Vitamin D plays important roles in the regulation of the hypothalamic–pituitary–thyroid axis, which has been indicated as the potential mechanism linking 25(OH)D levels with thyroid function [3,33]. The vitamin D receptor has been identified in murine thyrotrophic cells and follicular thyroid cells [33,45]. There was a strong molecular homology between the thyroid hormone and vitamin D receptor. Laboratory experiments showed that incubation of follicular thyroid cells with 1,25(OH)_2_D decreased the TSH-stimulated iodine uptake and inhibited cell growth [45]. Simultaneously, 1,25(OH)_2_D increased the thyrotropin-releasing hormone and induced TSH and thyrotropin releasing in vitro pituitary cells [46]. In addition, an animal study demonstrated that vitamin D supplementation decreased FT4 and increased the expression of the enzyme for the conversion of T4 to T3 deiodinase 2 (Dio2) in both the livers and brains of diabetic rats [47]. All the evidence suggested that vitamin D can regulate both central and peripheral levels and actions of thyroid hormones. And, more experiments are required to clarify the specific mechanisms.

There are several limitations in our study. First, as this is a cross-sectional study, the cause–effect relationship between vitamin D and impaired sensitivity to thyroid hormones is unclear and needs further exploration in randomized controlled trials and prospective studies. In addition, we did not collect data on sunlight exposure, physical activity, food intake, and parathormone, which might be important impact factors for 25(OH)D levels. Therefore, we could not determine whether the associations identified in this study were affected by these factors. However, the serum 25(OH)D tested in this study reflected the actual level of vitamin D within each individual determined by all the influencing factors. Moreover, the cut-off value of serum 25(OH)D for vitamin D deficiency is arbitrary, and some scientists suggested higher levels than 20 ng/mL as the thresholds based on the relationship between 25(OH)D concentrations and various diseases or disorders. Therefore, whether a 25(OH)D of 20 ng/mL is actually the best cut-off for identifying the association of vitamin D deficiency with impaired sensitivity to thyroid hormones is unclear and needs further investigation. Finally, our study was performed in Beijing, the biggest city in Northern China with a highly developed economy. Subjects in this study could well represent the urban adults in North China but not those in rural areas or in Southern China.

## 5. Conclusions

In conclusion, to our knowledge, this is the first study to explore the association of vitamin D with thyroid hormone sensitivity in an euthyroid adult Chinese population. We disclose the relationship between vitamin D deficiency and increased risks of impaired sensitivity to thyroid hormones, which is independent of multiple confounders. This finding indicated that vitamin D monitoring should be advocated, particularly in those with impaired sensitivity to thyroid hormones. The role of vitamin D supplementation in thyroid hormone sensitivity needs further investigation.

## Figures and Tables

**Table 1:** The clinical characteristics of the population.

Variables	Total Population	Vitamin D Sufficiency (≥20 ng/mL)	Vitamin D Deficiency (<20 ng/mL)	*p*
N	3143	1294	1849	NA
Age (years)	47.0 ± 11.9	47.5 ± 11.8	46.8 ± 12.0	0.105
Sex, men, n (%)	2012 (64.0%)	836 (64.6%)	1176 (63.6%)	0.571
BMI (kg/m^2^)	25.02 ± 3.57	24.92 ± 3.46	25.10 ± 3.65	0.123
SBP (mmHg)	125.36 ± 17.59	124.64 ± 17.86	125.86 ± 17.38	0.057
DBP (mmHg)	74.54 ± 11.71	74.26 ± 11.86	74.74 ± 11.60	0.264
ALT (U/L) *	21.0 (16.0–31.0)	21.0 (16.0–30.0)	21.0 (16.0–31.0)	0.876
AST (U/L) *	22.0 (19.0–26.0)	22.0 (19.0–26.0)	22.0 (19.0–26.0)	0.151
Urea (mmol/L)	5.32 ± 1.27	5.37 ± 1.26	5.28 ± 1.27	0.053
Cr (umol/L)	67.19 ± 13.33	68.26 ± 13.49	66.44 ± 13.16	<0.001
UA (umol/L)	361.82 ± 89.64	366.72 ± 87.58	358.39 ± 90.92	0.010
Ca (mmol/L)	2.39 ± 0.09	2.40 ± 0.09	2.39 ± 0.09	0.011
P (mmol/L)	1.15 ± 0.22	1.15 ± 0.16	1.15 ± 0.25	0.560
25(OH)D (ng/mL)	19.30 ± 7.39	26.18 ± 6.00	14.50 ± 3.56	<0.001
TC (mmol/L)	5.08 ± 0.96	5.05 ± 0.94	5.10 ± 0.96	0.119
TG (mmol/L) *	1.38 (0.97–2.01)	1.33 (0.96–1.89)	1.42 (0.99–2.10)	<0.001
HDL-C (mmol/L)	1.29 ± 0.34	1.31 ± 0.34	1.28 ± 0.33	0.014
LDL-C (mmol/L)	3.10 ± 0.86	3.07 ± 0.85	3.12 ± 0.87	0.123
Non-HDL-C (mmol/L)	3.79 ± 0.92	3.74 ± 0.91	3.83 ± 0.93	0.012
RC (mmol/L) *	0.60 (0.39–0.86)	0.60 (0.38–0.86)	0.61 (0.39–0.86)	0.729
AIP	0.063 ± 0.306	0.037 ± 0.295	0.081 ± 0.313	<0.001
Lp(a) (mmol/L) *	12.50 (7.20–24.40)	12.60 (7.30–24.03)	12.40 (7.10–24.60)	0.889
Glucose (mmol/L) *	4.94 (4.59–5.38)	4.94 (4.59–5.38)	4.94 (4.59–5.38)	0.964
HbA1c (%) *	5.5 (5.3–5.8)	5.5 (5.3–5.6)	5.5 (5.3–5.8)	0.349
Insulin (mIU/L) *	8.1 (5.7–11.5)	7.7 (5.6–11.2)	8.4 (5.8–11.7)	0.007
HOMA-IR *	1.80 (1.22–2.73)	1.74 (1.19–2.63)	1.86 (1.24–2.80)	0.018
HOMA-β *	113.51 (74.56–171.12)	109.31 (72.30–164.09)	116.33 (75.50–175.51)	0.028
HOMA-ISI *	0.025 (0.017–0.036)	0.026 (0.017–0.037)	0.024 (0.016–0.036)	0.018
TyG (mg/dL)^2^	8.68 ± 0.63	8.63 ± 0.60	8.71 ± 0.65	<0.001
FT3 (pg/mL)	3.28 ± 0.35	3.26 ± 0.34	3.29 ± 0.35	0.009
FT4 (ng/dL)	1.29 ± 0.15	1.27 ± 0.15	1.29 ± 0.16	<0.001
TSH (uIU/mL)	2.08 ± 0.93	2.00 ± 0.90	2.14 ± 0.94	<0.001
Anti-TG positivity, n (%)	294 (9.4%)	109 (8.4%)	185 (10.0%)	0.136
Anti-TPO positivity, n (%)	284 (9.0%)	111 (8.6%)	173 (9.4%)	0.487
TFQI	−0.002 ± 0.382	−0.053 ± 0.388	0.033 ± 0.375	<0.001
PTFQI	0.053 ± 0.386	0.002 ± 0.391	0.088 ± 0.378	<0.001
TSHI	2.86 ± 0.49	2.80 ± 0.49	2.90 ± 0.49	<0.001
TT4RI	34.19 ± 15.12	32.58 ± 14.80	35.31 ± 15.23	<0.001
FT3/FT4	0.308 ± 0.040	0.309 ± 0.039	0.308 ± 0.041	0.281

* Non-normally distributed continuous variables were compared after natural log transformation. Continuous variables were analyzed by Student’s *t*-test. Categorical variables were compared with Fischer’s exact tests. Abbreviations: NA, not applicable; BMI, body mass index; SBP, systolic blood pressure; DBP, diastolic blood pressure; ALT, alanine aminotransferase; AST, aspartate aminotransferase; Cr, creatinine; UA, uric acid; 25(OH)D, 25-hydroxyvitamin D; TC, total cholesterol; TG, triglyceride; HDL-C, high-density lipoprotein cholesterol; LDL-C, low-density lipoprotein cholesterol; RC, remnant cholesterol; AIP, atherogenic index of plasma; Lp(a), lipoprotein a; Ca, calcium; P, phosphorus; HbA1c, Hemoglobin A1c; HOMA-IR, homeostatic model for insulin resistance; HOMA-β, homeostatic model β; HOMA-ISI, homeostatic model for insulin sensitivity index; TyG, triglyceride-glucose index; FT3, free thyroxine; FT4, free thyrotropin; TSH, thyroid-stimulating hormone; Anti-TG, antithyroglobulin; Anti-TPO, antithyroidperoxidase; TFQI, thyroid feedback quartile-based index; PTFQI, parametric thyroid feedback quantile-based index; TSHI, TSH index; and TT4RI, thyrotropin thyroxine resistance index.

**Table 2:** The relationship between 25(OH)D and thyroid-associated variables.

Variables	Unadjusted		Adjusted 1		Adjusted 2	
	r	*p*	r	*p*	r	*p*
FT3 (pg/mL)	−0.041	0.022	−0.040	0.027	−0.036	0.042
FT4 (ng/dL)	−0.049	0.006	−0.038	0.032	−0.041	0.023
TSH (uIU/mL)	−0.090	<0.001	−0.077	<0.001	−0.083	<0.001
TFQI	−0.107	<0.001	−0.086	<0.001	−0.094	<0.001
PTFQI	−0.106	<0.001	−0.086	<0.001	−0.093	<0.001
TSHI	−0.109	<0.001	−0.089	<0.001	−0.096	<0.001
TT4RI	−0.103	<0.001	−0.085	<0.001	−0.092	<0.001
FT3/FT4	0.012	0.516	0.005	0.784	0.009	0.595

Data were analyzed by Pearson’s and partial correlation coefficients. Adjusted 1: age, sex, and BMI; Adjusted 2: age, sex, BMI, DBP, TG, glucose, ALT, Cr, and UA. Abbreviations: 25(OH)D, 25-hydroxyvitamin D; FT3, free triiodothyronine; FT4, free thyrotropin; TSH, thyroid-stimulating hormone; TFQI, thyroid feedback quartile-based index; PTFQI, parametric thyroid feedback quantile-based index; TSHI, TSH index; TT4RI, thyrotropin thyroxine resistance index; BMI, body mass index; DBP, diastolic blood pressure; TG, triglyceride; ALT, alanine aminotransferase; Cr, creatinine; and UA, uric acid.

**Table 3:** Logistic regression analysis of the relationship between vitamin D deficiency and impaired sensitivity to thyroid hormones.

Impaired Sensitivity to Thyroid Hormones	Model 1		Model 2		Model 3	
	OR (95%CI)	*p*	OR (95%CI)	*p*	OR (95%CI)	*p*
TFQI	1.66 (1.44, 1.92)	<0.001	1.65 (1.43, 1,91)	<0.001	1.68 (1.45, 1.95)	<0.001
PTFQI	1.67 (1.44, 1.93)	<0.001	1.66 (1.44, 1.93)	<0.001	1.70 (1.46, 1.97)	<0.001
TSHI	1.62 (1.41, 1.87)	<0.001	1.61 (1.39, 1.86)	<0.001	1.66 (1.43, 1.92)	<0.001
TT4RI	1.48 (1.28, 1.70)	<0.001	1.46 (1.26, 1.69)	<0.001	1.50 (1.30, 1.74)	<0.001
FT3/FT4	1.11 (0.96, 1.28)	0.164	1.11 (0.96, 1.29)	0.148	1.14 (0.98, 1.32)	0.083

Data were analyzed by logistic regression analysis. Model 1: unadjusted; Model 2: adjusted for age, sex, and BMI; Model 3: adjusted for age, sex, BMI, DBP, TG, glucose, ALT, Cr, and UA. Abbreviations: OR, odds ratio; CI, confidence interval; TFQI, thyroid feedback quartile-based index; PTFQI, parametric thyroid feedback quantile-based index; TSH, thyroid-stimulating hormone; TSHI, TSH index; TT4RI, thyrotropin thyroxine resistance index; FT3, free triiodothyronine; FT4, free thyrotropin; BMI, body mass index; DBP, diastolic blood pressure; TG, triglyceride; ALT, alanine aminotransferase; Cr, creatinine; and UA, uric acid.

**Table 4:** Logistic regression analysis of the relationship between vitamin D deficiency and impaired sensitivity to thyroid hormones in males and females after adjusting for age, BMI, DBP, TG, glucose, ALT, Cr, and UA.

Impaired Sensitivity to Thyroid Hormone	Men (N = 2012)		Women (N = 1131)		*p* for Interaction
	OR (95%CI)	*p*	OR (95%CI)	*p*	
TFQI	1.25 (1.04, 1.50)	0.020	2.84 (2.19, 3.67)	<0.001	<0.001
PTFQI	1.25 (1.04, 1.51)	0.016	2.92 (2.25, 3.78)	<0.001	<0.001
TSHI	1.24 (1.04, 1.49)	0.020	2.86 (2.21, 3.70)	<0.001	<0.001
TT4RI	1.18 (0.98, 1.41)	0.083	2.40 (1.86, 3.10)	<0.001	<0.001
FT3/FT4	1.03 (0.86, 1.24)	0.765	1.43 (1.11, 1.83)	0.006	<0.001

Data were analyzed by logistic regression analysis. Abbreviations: BMI, body mass index; DBP, diastolic blood pressure; TG, triglyceride; ALT, alanine aminotransferase; Cr, creatinine; UA, uric acid; OR, odds ratio; CI, confidence interval; TFQI, thyroid feedback quartile-based index; PTFQI, parametric thyroid feedback quantile-based index; TSH, thyroid-stimulating hormone; TSHI, TSH index; TT4RI, thyrotropin thyroxine resistance index; FT3, free triiodothyronine; and FT4, free thyrotropin.

## Data Availability

The data presented in this study are available on request from the corresponding author.

## Supplementary Materials

The following supporting information can be downloaded at: https://www.mdpi.com/article/10.3390/nu15173697/s1, Figure S1. Flowchart of this study. Figure S2. The proportions of participants with impaired sensitivity to thyroid hormones in men and women in the vitamin D sufficiency group and the vitamin D deficiency group. Data was analyzed by Fisher’s exact test. (A) TFQI; (B) PTFQI; (C) TSHI; (D)TT4RI; and (E) FT3/FT4. TFQI, thyroid feedback quartile-based index; PTFQI, parametric thyroid feedback quantile-based index; TSH, thy-roid-stimulating hormone; TSHI, TSH index; TT4RI, thyrotropin thyroxine resistance index; FT3, free triiodothyronine; and FT4, free thyrotropin. Table S1. The clinical characteristics in men and women. Table S2. The relationship between 25(OH)D levels and thyroid-associated variables after adjusting for age, BMI, DBP, TG, glucose, ALT, Cr, and UA in men and women.
