# Supplementary material for: Vitamin D Deficiency Is Associated with Impaired Sensitivity to Thyroid Hormones in Euthyroid Adults
Source: Nutrients. 2023 Aug 24;15(17):3697. doi: 10.3390/nu15173697 (PMC10490158; doi:10.3390/nu15173697)
Supplement: Supplementary file 1 [file nutrients-15-03697-s001.zip › nutrients-2565796-supplementary.pdf]

nutrients

MDPI

Article

Vitamin D Deficiency Is Associated with Impaired Sensitivity to Thyroid Hormones in Euthyroid Adults

Liyuan Zhou [1,†], Ying Wang [2,†], Jingru Su [1], Yu An [1], Jia Liu [1] and Guang Wang [1,*]

1 Department of Endocrinology, Beijing Chao-Yang Hospital, Capital Medical University, Beijing 100020, China
2 Medical Examination Center, Beijing Chao-Yang Hospital, Capital Medical University, Beijing 100020, China
* Correspondence: wangguang@bjcyh.com
† These authors contributed equally to this work.

Table S1. The clinical characteristics in men and women.

Variables	Vitamin D sufficiency (≥20ng/mL)		Vitamin D deficiency (<20ng/mL)		*p*
	Men	Women	Men	Women	
N	836	458	1176	673	NA
Age (years)	46.5±11.0	49.3±12.8[†]	44.3±11.7[&]	51.0±11.4[#]	<0.001
BMI (kg/m²)	25.74±3.11	23.38±3.55[†]	25.82±3.46	23.84±3.64[#]	<0.001
SBP (mmHg)	126.73±16.52	120.84±19.55[†]	126.66±15.78	124.47±19.79[#, $]	<0.001
DBP (mmHg)	76.70±11.84	69.81±10.54[†]	76.64±11.24	71.42±11.49[#]	<0.001
ALT (U/L)*	24.0(18.0-34.0)	17.0(14.0-22.0)[†]	25.0(18.0-35.0)	17.0(14.0-23.0)[#]	<0.001
AST (U/L)*	23.0(20.0-27.0)	21.0(18.0-24.0)[†]	22.0(19.0-27.0)	21.0(18.0-24.0)[#]	<0.001
Urea (mmol/L)	5.54±1.26	5.07±1.20[†]	5.43±1.20	5.03±1.34[#]	<0.001
Cr (umol/L)	74.85±11.18	56.23±7.89[†]	73.08±10.03[&]	54.82±9.39[#]	<0.001
UA (umol/L)	400.15±79.55	305.68±66.01[†]	397.31±80.39	290.37±64.40[#, $]	<0.001
Ca (mmol/L)	2.40±0.09	2.39±0.09	2.40±0.09	2.37±0.08[#, $]	<0.001
P (mmol/L)	1.10±0.14	1.23±0.15[†]	1.10±0.16	1.23±0.35[#]	<0.001
25(OH)D (ng/mL)	26.20±5.93	26.15±6.15	14.84±3.19[&]	13.90±3.56[#, $]	<0.001
TC (mmol/L)	5.00±0.92	5.14±0.98	5.02±0.92	5.24±1.12[#]	<0.001
TG (mmol/L)*	1.44(1.03-2.04)	1.10(0.83-1.63)[†]	1.56(1.06-2.29)[&]	1.23(0.90-1.71)[#]	<0.001
HDL-C (mmol/L)	1.20±0.27	1.50±0.38[†]	1.18±0.27	1.45±0.36[#]	<0.001
LDL-C (mmol/L)	3.11±0.83	3.01±0.89	3.12±0.84	3.13±0.92	0.121
Non-HDL-C (mmol/L)	3.80±0.87	3.64±0.96[†]	3.84±0.90	3.80±0.98[$]	0.001
RC (mmol/L)*	0.61(0.39-0.88)	0.59(0.37-0.80)	0.61(0.39-0.88)	0.62(0.40-0.83)	0.334
AIP	0.107±0.279[&]	-0.092±0.278[†]	0.152±0.305	-0.043±0.287[#, $]	<0.001
Lp(a) (mmol/L)*	11.65(6.63-21.10)	14.80(8.28-29.15)[†]	11.10(6.50-22.40)	15.20(8.70-29.00)	<0.001
Glucose (mmol/L)*	4.98(4.59-5.45)	4.87(4.57-5.29)[†]	4.96(4.60-5.39)	4.92(4.58-5.34)	0.004
HbA1c (%)*	5.5(5.3-5.8)	5.6(5.3-5.9)	5.5(5.3-5.8)	5.6(5.3-5.8)	0.836
Insulin (mIU/L)*	7.9(5.7-11.3)	7.4(5.3-10.8)	8.7(5.9-12.2)[&]	7.9(5.5-10.8)[#]	<0.001
HOMA-IR*	1.80(1.25-2.72)	1.67(1.11-2.40)	1.95(1.28-2.96)	1.72(1.17-2.50)[#]	<0.001
HOMA-β*	109.40(70.82-166.67)	109.23(75.51-160.65)	117.75(75.00-184.88)	111.26(76.64-164.91)	0.057
HOMA-ISI*	0.025(0.016-0.036)	0.027(0.019-0.040)	0.023(0.016-0.035)	0.026(0.018-0.038)[#]	<0.001

TyG (mg/dL)[2]	8.73±0.60	8.45±0.55[†]	8.81±0.67[&]	8.55±0.58[#]	<0.001
FT3 (pg/mL)	3.37±0.31	3.05±0.28[†]	3.41±0.32	3.10±0.32[#]	<0.001
FT4 (ng/dL)	1.30±0.15	1.23±0.14[†]	1.32±0.16	1.25±0.14[#, $]	<0.001
TSH (uIU/mL)	1.90±0.84	2.18±0.98[†]	1.93±0.82	2.51±1.02[#, $]	<0.001
Anti-TG positivity, n (%)	43(5.1%)	66(14.4%)[†]	71(6.0%)	114(16.9%)*	<0.001
Anti-TPO positivity, n (%)	45(5.4%)	66(14.4%)[†]	74(6.3%)	99(14.7%)*	<0.001
TFQI	-0.032±0.382	-0.090±0.395[†]	0.014±0.386[&]	0.068±0.352[#, $]	<0.001
PTFQI	0.023±0.386	-0.036±0.398[†]	0.068±0.389[&]	0.123±0.355[#, $]	<0.001
TSHI	2.80±0.48	2.80±0.51	2.85±0.49	3.00±0.47[#, $]	<0.001
TT4RI	31.64±14.26	34.30±15.62[†]	32.57±14.02	40.09±16.07[#, $]	<0.001
FT3/FT4	0.314±0.038	0.301±0.039[†]	0.313±0.041	0.299±0.039[#]	<0.001

*Non-normally distributed continuous variables were compared after natural log transformation. Continuous variables were analyzed by one-way ANOVA with tukey's post hoc test. Categorical variables were compared with Fischer's exact tests. [†]: significant difference between men and women in the vitamin D sufficiency group; [#]: significant difference between men and women in the vitamin D deficiency group; [&]: significant difference between the vitamin D sufficiency group and the vitamin D deficiency group in men; [$]: significant difference between the vitamin D sufficiency group and the vitamin D deficiency group in women. Abbreviations: NA, not applicable; BMI, body mass index; SBP, systolic blood pressure; DBP, diastolic blood pressure; ALT, alanine aminotransferase; AST, aspartate aminotransferase; Cr, creatinine; UA, uric acid; 25(OH)D, 25-hydroxyvitamin D; TC, total cholesterol; TG, triglyceride; HDL-C, high-density lipoprotein cholesterol; LDL-C, low-density lipoprotein cholesterol; RC, remnant cholesterol; AIP, atherogenic index of plasma; Lp(a), lipoprotein a; Ca, calcium; P, phosphorus; HbA1c, Hemoglobin A1c; HOMA-IR, homeostatic model for insulin resistance; HOMA-β, homeostatic model β; HOMA-ISI, homeostatic model for insulin sensitivity index; TyG, triglyceride-glucose index; FT3, free thyroxine; FT4, free thyrotropin; TSH, thyroid-stimulating hormone; Anti-TG, antithyroglobulin; Anti-TPO, antithyroidperoxidase. TFQI, thyroid feedback quartile-based index; PTFQI, parametric thyroid feedback quantile-based index; TSHI, TSH index; and TT4RI, thyrotropin thyroxine resistance index.

Table S2. The relationship between 25(OH)D levels and thyroid-associated variables after adjusting for age, BMI, DBP, TG, glucose, ALT, Cr, and UA in men and women.

**Variables**	**Men**		**Women**	
	**r**	***p***	**r**	***p***
FT3 (pg/mL)	--0.001	0.977	-0.068	0.023
FT4 (ng/dL)	-0.001	0.958	-0.101	0.001
TSH (uIU/mL)	-0.038	0.088	-0.144	<0.001
TFQI	-0.027	0.220	-0.198	<0.001
PTFQI	-0.028	0.214	-0.198	<0.001
TSHI	-0.040	0.073	-0.183	<0.001
TT4RI	-0.039	0.082	-0.167	<0.001
FT3/FT4	-0.002	0.920	0.041	0.167

Data was analyzed by Pearson's and partial correlation coefficients. Abbreviations: 25(OH)D, 25-hydroxyvitamin D; FT3, free triiodothyronine; FT4, free thyrotropin; TSH, thyroid-stimulating hormone; TFQI, thyroid feedback quartile-based index; PTFQI, parametric thyroid feedback quantile-based index; TSHI, TSH index; TT4RI, thyrotropin thyroxine resistance index; BMI, body mass index; DBP, diastolic blood pressure; TG, triglyceride; ALT, alanine aminotransferase; Cr, creatinine; and UA, uric acid.

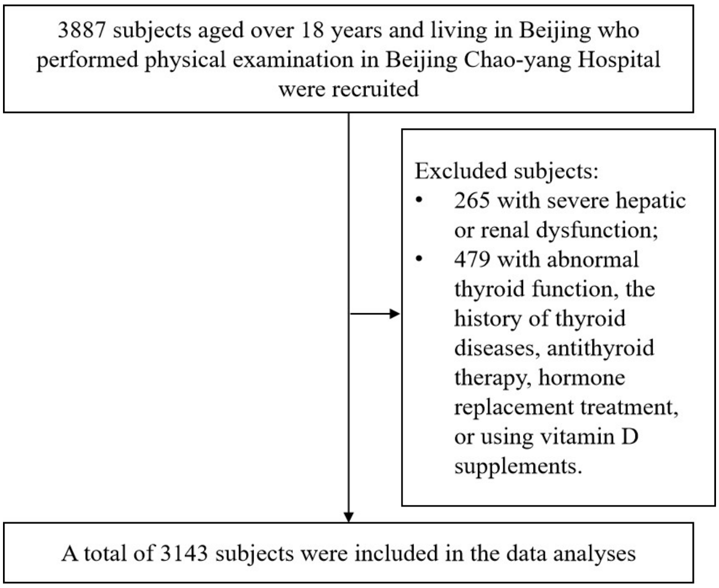

Figure S1. Flowchart of this study.

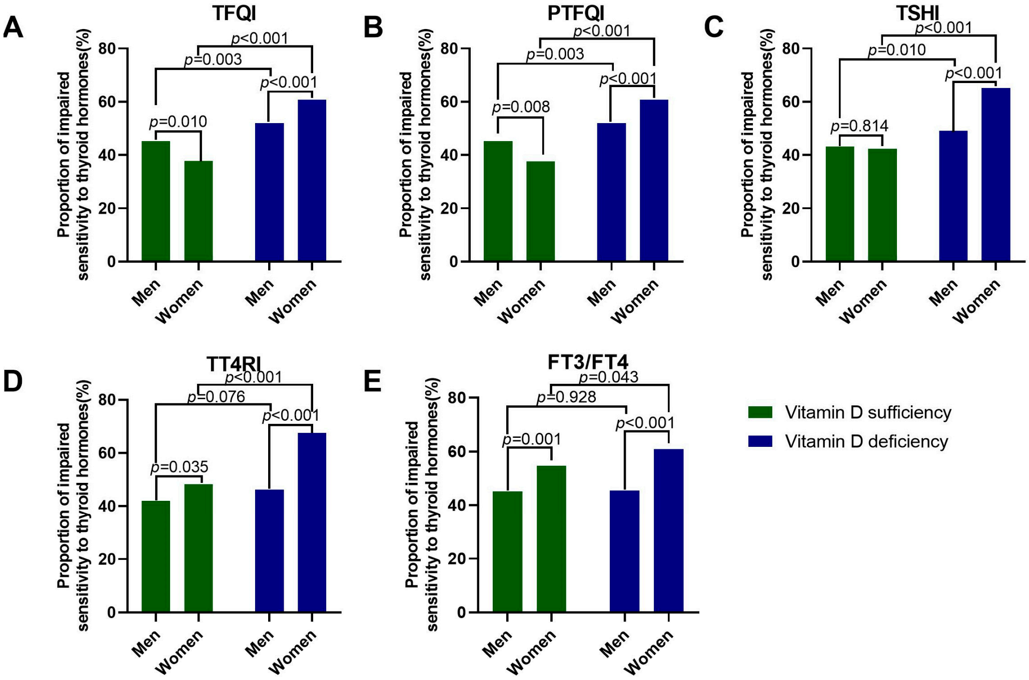

Figure S2. The proportions of participants with impaired sensitivity to thyroid hormones in men and women in the vitamin D sufficiency group and the vitamin D deficiency group. Data was analyzed by Fisher's exact test. (A) TFQI; (B) PTFQI; (C) TSHI; (D)TT4RI; and (E) FT3/FT4. TFQI, thyroid feedback quartile-based index; PTFQI, parametric thyroid feedback quantile-based index; TSH, thyroid-stimulating hormone; TSHI, TSH index; TT4RI, thyrotropin thyroxine resistance index; FT3, free triiodothyronine; and FT4, free thyrotropin.
